# Standardized orthotopic xenografts in zebrafish reveal glioma cell-line-specific characteristics and tumor cell heterogeneity

**DOI:** 10.1242/dmm.022921

**Published:** 2016-02-01

**Authors:** Alessandra M. Welker, Brian D. Jaros, Vinay K. Puduvalli, Jaime Imitola, Balveen Kaur, Christine E. Beattie

**Affiliations:** 1 Department of Neuroscience, The Ohio State University College of Medicine, Columbus, OH 43210, USA; 2 Department of Neurosurgery, The Ohio State University College of Medicine, Columbus, OH 43210, USA; 3 Department of Neurology, The Ohio State University College of Medicine, Columbus, OH 43210, USA

**Keywords:** GBM9 neurospheres, Glial fibrillary acidic protein, Glioblastoma, Sox2, X12 cells, Temozolomide

## Abstract

Glioblastoma (GBM) is a deadly brain cancer, for which few effective drug treatments are available. Several studies have used zebrafish models to study GBM, but a standardized approach to modeling GBM in zebrafish was lacking to date, preventing comparison of data across studies. Here, we describe a new, standardized orthotopic xenotransplant model of GBM in zebrafish. Dose-response survival assays were used to define the optimal number of cells for tumor formation. Techniques to measure tumor burden and cell spread within the brain over real time were optimized using mouse neural stem cells as control transplants. Applying this standardized approach, we transplanted two patient-derived GBM cell lines, serum-grown adherent cells and neurospheres, into the midbrain region of embryonic zebrafish and analyzed transplanted larvae over time. Progressive brain tumor growth and premature larval death were observed using both cell lines; however, fewer transplanted neurosphere cells were needed for tumor growth and lethality. Tumors were heterogeneous, containing both cells expressing stem cell markers and cells expressing markers of differentiation. A small proportion of transplanted neurosphere cells expressed glial fibrillary acidic protein (GFAP) or vimentin, markers of more differentiated cells, but this number increased significantly during tumor growth, indicating that these cells undergo differentiation *in vivo*. By contrast, most serum-grown adherent cells expressed GFAP and vimentin at the earliest times examined post-transplant. Both cell types produced brain tumors that contained Sox2^+^ cells, indicative of tumor stem cells. Transplanted larvae were treated with currently used GBM therapeutics, temozolomide or bortezomib, and this resulted in a reduction in tumor volume *in vivo* and an increase in survival. The standardized model reported here facilitates robust and reproducible analysis of glioblastoma tumor cells in real time and provides a platform for drug screening.

## Abstract (precis)

**Summary:** This zebrafish xenotransplant model of glioblastoma enables *in vivo* imaging of tumor cells and rapid screening for anti-glioma agents. It provides standardization of a model that is easily replicated across laboratories.

## INTRODUCTION

Glioblastoma (GBM) is the most aggressive primary brain cancer and is classified as grade IV by the World Health Organization. Patient survival time after diagnosis is 12-18 months, and the 5 year survival rate is only 10%, with limited therapeutic options (Ostrom et al., 2014). Treatment for GBM includes surgical resection, followed by concurrent radiotherapy and chemotherapy with temozolomide (a DNA-alkylating agent). Even with these therapies, prognosis is poor and the vast majority of tumors recur within 6 months of resection. Histological characterization of GBM reveals areas of hypoxia, necrosis, angiogenesis and vascular hyperproliferation (Agnihotri et al., 2013; Popova et al., 2014). The highly infiltrative nature of GBM cells, specifically in brain tissue, makes them even harder to resect and treat (Ananthakrishnan and Ehrlicher, 2007; Kwiatkowska et al., 2012; Hu et al., 2012). Thus, there is an urgent need to identify new drug candidates to target this fatal disease.

Current murine models of GBM include genetic and orthotopic transplant models. Strengths of murine genetic models include an intact immune system and endogenous tumor formation. Orthotopic transplant (transplant of brain tumor cells into brain tissue) murine models use primary patient-derived glioma cells implanted into the cortex of immunocompromised mice (Dinca et al., 2007). These models have been used extensively to evaluate the efficacy and toxicity of new therapeutics and are currently US Food and Drug Administration (FDA)-approved models for the development of preclinical therapeutics crucial for investigational new drug status. Mouse models have also been widely used for understanding tumor biology and for therapeutic drug development; however, they can be expensive and cannot be used for large drug-screening approaches. Bioluminescence and magnetic resonance imaging are common methods of gross tumor detection in mice (Chen et al., 2013); nonetheless, it is difficult in mammalian models to obtain the cellular resolution necessary to analyze tumor cell migration and formation *in vivo*.

The zebrafish is a freshwater vertebrate that has been used for decades to understand vertebrate development and genetics (Mushtaq et al., 2013). More recently, zebrafish have been exploited to analyze tumor development and for chemical screens (Peal et al., 2010; Lally et al., 2007), leading to the identification of several candidate therapeutics for melanoma and leukemia that are currently being evaluated in patients (NCT02354417, NCT01611675 and NCT01512251; White et al., 2013; Konantz et al., 2012). Glioma cells have previously been transplanted into zebrafish (Vittori et al., 2015). In several studies, serum-grown adherent glioma cell lines were transplanted into the yolk to analyze angiogenesis and tumor growth and showed that these cells formed tumors on the yolk, promoted angiogenesis and were responsive to therapeutics (Geiger et al., 2008; Zhao et al., 2009; Lally et al., 2007; Yang et al., 2013). Recently, a number of groups have transplanted glioma cells into the zebrafish brain. Lal et al. (2012) analyzed angiogenesis and invasion using a serum-grown adherent line of glioma cells, and Rampazzo et al. (2013) analyzed the role of wnt signaling in glioma cell differentiation. In another study, thousands of purified glioma stem cells were transplanted into larval zebrafish for the purpose of drug screening, as an interim model between cell culture and mice (Kitambi et al., 2014). Lastly, an orthotopic model of pediatric brain tumors and glioma was generated by intranasal injection into adult immunocompromised zebrafish. Tumors grew and expressed genes typical of the tumor type and were responsive to therapeutics (Eden et al., 2014). These studies show the potential to use zebrafish as a model for glioma. However, no study to date has fully characterized how different glioma cells grow and behave in the zebrafish brain nor have these experiments been standardized so that they can be used for drug screens across studies. Moreover, it is not known how glioma cells change over time as they form tumors when transplanted into mouse or zebrafish brains. Thus, having a standardized model in a complementary vertebrate model system will significantly benefit glioma research.

Here, we describe the standardization, characterization and utility of an orthotopic zebrafish model of glioma that enables *in vivo*, real-time imaging of individual tumor cells and enables drug screening for glioma therapy. We show that both neurospheres and serum-grown adherent cell tumors express markers of stem cells and differentiated cells, but the cellular characteristics of neurospheres change more dramatically during tumor development *in vivo*. In addition, varying the number of transplanted cells reveals that neurospheres are more lethal than serum-grown adherent cells. This thorough characterization of tumor formation in zebrafish will allow for standardization of these transplants and will serve as a valuable model for investigating glioma biology and for rapid and less expensive whole-animal drug testing.

## RESULTS

### Intracranial transplant of glioblastoma cells in zebrafish causes lethality

**Fig. 1.**
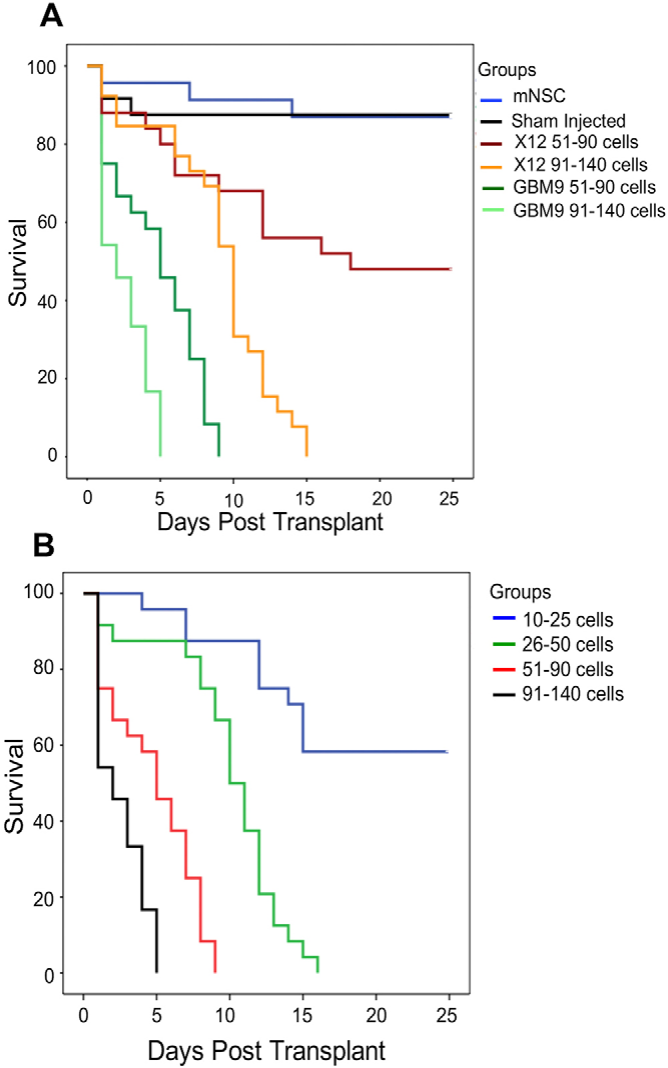
**Survival of zebrafish xenotransplants.** (A) Control animals were transplanted with ∼50 mouse neural stem cells (mNSCs; blue line) or sham injected with 1-2 nl of HBSS (black line), with 87.5% survival in both groups. Animals transplanted with 51-90 GBM9 cells (dark green line) had a median survival of 5±1.0 dpt, compared with 2 dpt for animals transplanted with 91-140 GBM9 cells (light green line). Animals transplanted with 51-90 X12 cells (red line) engrafted and formed tumors only 50% of the time, with a median survival of 18 dpt, whereas animals transplanted with 91-140 X12 cells (orange line) had a median survival of 10±0.5 dpt. *n*=100 animals per group for GBM9 and X12 groups and *n*=24 for mNSCs and sham-injected groups. *P*<0.0001 for all tumor xenotransplants compared with mNSC or sham. (B) GBM9 xenotransplants were scored at 1 dpt for the number of engrafted tumor cells; 10-25 cells (blue line), 26-50 cells (green line) with a median survival of 10±0.7 dpt, 51-90 cells (red line) with a median survival of 5±1.0 dpt, or 91-140 cells (black line) with a median survival of 2 days. *n*=24 animals per group. *P*<0.0001 for GBM9 transplants of 25 cells or greater compared with those receiving <20 cells.

To generate a model of glioblastoma in zebrafish, we designed an orthotopic xenotransplant (human brain cells into zebrafish brain) approach. To ensure consistency, we used the midbrain-hindbrain boundary as a landmark for all cell transplants in 36 hour postfertilization (hpf) wild-type embryos. We evaluated two different patient-derived glioma cells lines expressing green fluorescent protein (GFP), serum-grown adherent X12 cells and GBM9 neurospheres (Williams et al., 2011; Godlewski et al., 2008; Wojton et al., 2014; Giannini, 2005). Two different cell quantities, 51-90 cells and 91-140 cells, were transplanted for each cell line. We also transplanted control mouse neural stem cells (mNSCs), in addition to sham injections. Kaplan–Meier survival curves revealed that sham injection or mNSC transplantion had only a minor affect on survival, with 87.5% survival for both conditions (Fig. 1A; Table S1). Transplantation of both X12 and GBM9 cells had a negative effect on survival, but approximately twice as many X12 cells (91-140) were needed compared with GBM9 cells (51-90) to cause 100% lethality. Even at this dose, the median survival time of fish transplanted with 51-90 GBM9 cells was 5±1 days post-transplant (dpt) and for 91-140 X12 cells it was 10.0±0.5 dpt (Fig. 1A; *P*<0.0001; Table S1). To investigate further the effect of GBM9 cells, which had a larger affect on survival, we performed a cell-dilution analysis (Fig. 1B; Table S1). We found that ∼60% of fish transplanted with 10-25 cells survived. Analysis at 25 dpt revealed few GFP^+^ cells, suggesting that this low number of cells did not engraft to form tumors. Transplanting more than 25 cells resulted in 100% lethality. The median survival for 26-50 cells was 10.0±0.7 dpt and for 51-90 cells 5±1.0 dpt. Transplanting more than 91 cells caused rapid death, with a median survival of 2 dpt. These data show that both serum-grown and neurosphere human GBM cell lines cause lethality in zebrafish in a dose-dependent manner and that the GBM9 neurospheres are more lethal. We also observed statistically significant movement defects in these animals at 5 and 10 dpt (Fig. S1). To standardize these studies, ∼50 GBM9 or ∼100 X12 cells were transplanted in all subsequent experiments.

### *In vivo* imaging of xenotransplants reveals tumor growth over time

**Fig. 2.**
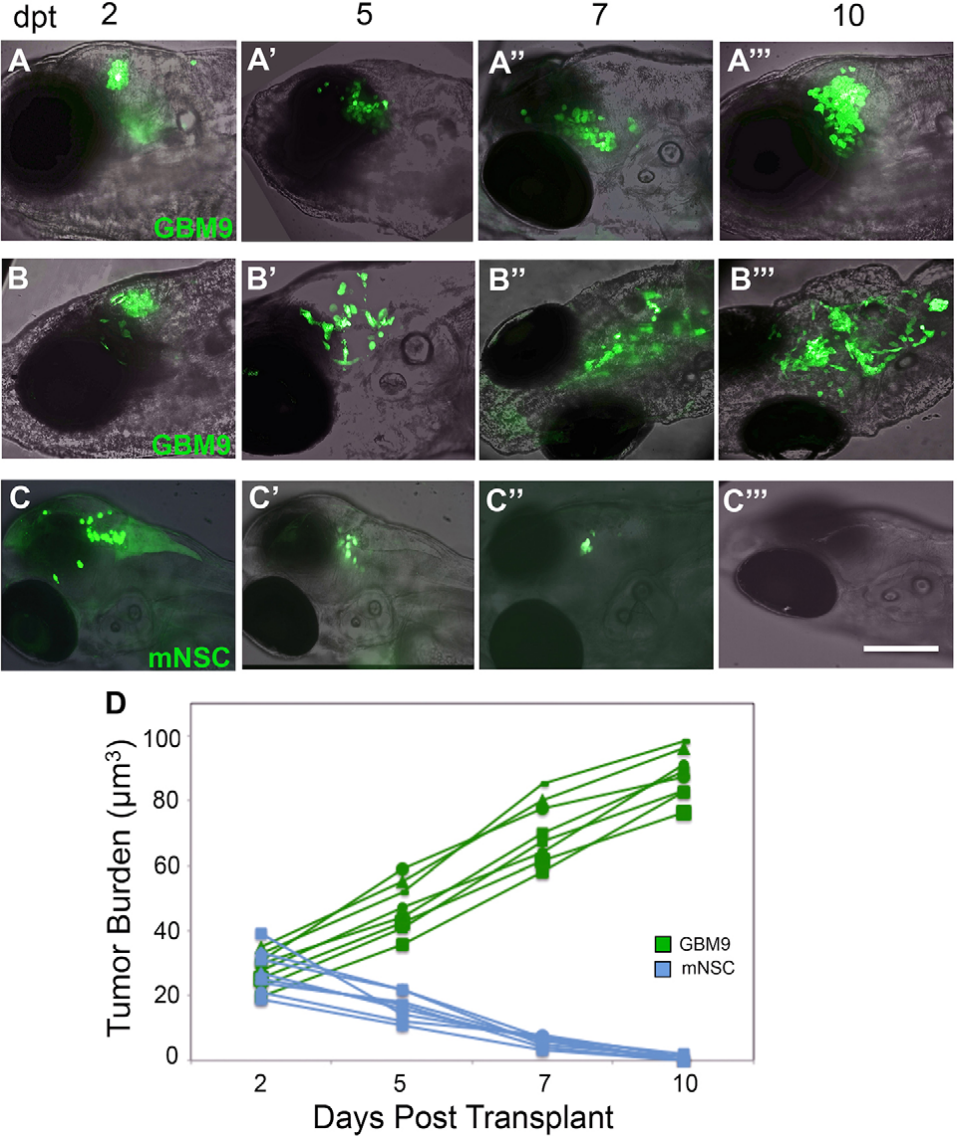
**Analysis of tumor burden in live animals over time.** Confocal images superimposed on bright field (anterior to the left) of two representative *casper* zebrafish transplanted with 50-75 GBM9 cells (A-A‴,B-B‴) and a *casper* animal transplanted with control mNSC cells (C-C‴) imaged at 2 (A,B,C), 5 (A′,B′,C′), 7 (A″,B″,C″) and 10 (A‴,B‴,C‴) dpt. Examples of a compact (A-A‴) and diffuse tumor (B-B‴) are shown. (D) Tumor burden were quantified using volume measurements of florescence in micrometers cubed. Approximately 50-75 GBM9 cells (green lines) and ∼50 mNSC cells (blue lines) were transplanted and followed over time in the same animal. *n*=8 animals per group. Scale bar: 50 μm.

We next addressed how the glioblastoma cells were behaving over time in the brain environment and focused on the more aggressive GBM9 cells. For these experiments we used *casper* zebrafish, which lack pigment genes in iridophores and melanocytes, resulting in optically transparent animals that are excellent for *in vivo* imaging (White et al., 2008). Using confocal microscopy, we observed GBM9 cells forming tumors and cells spreading throughout the brain. The same fish were imaged over 2, 5, 7 and 10 dpt, and representative images from three animals are shown in Fig. 2. Fish 1 (Fig. 2A-A‴) and fish 2 (Fig. 2B-B‴) contained GBM9 cells, and fish 3 (Fig. 2C-C‴) was transplanted with control mNSCs. The tumor burden was quantified over time by collecting a confocal *z*-series and determining cell volume using Metamorph software (see Materials and Methods). This revealed a time-dependent linear increase of tumor burden in GBM9-transplanted animals compared with control mNSC cell transplants (Fig. 2D). In contrast to GBM9 cells, imaging mNSCs cells over time revealed no tumor growth or cell migration, and no GFP^+^ cells were present at the end of the study (Fig. 2C-C‴,D).

**Fig. 3.**
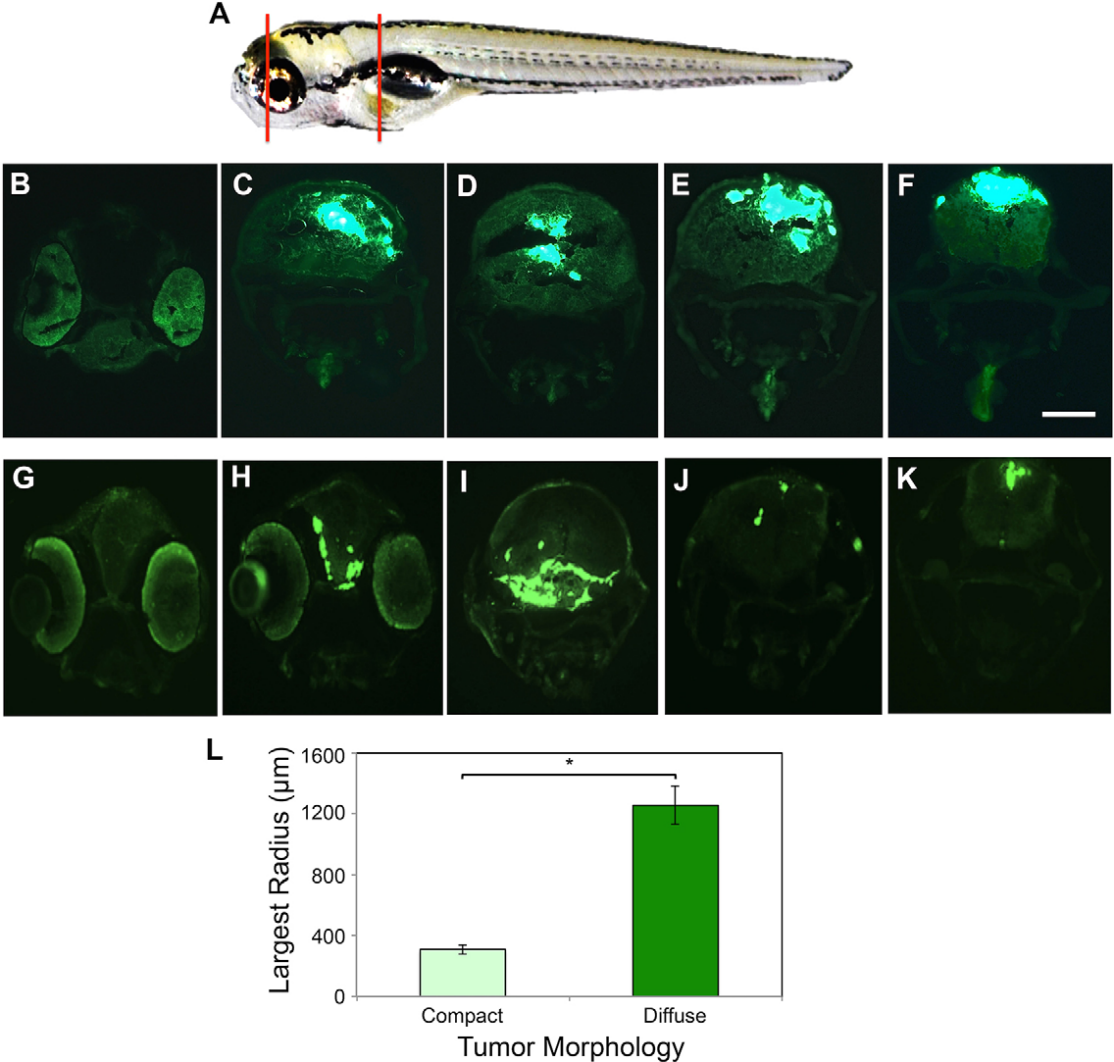
**GBM9 tumor cells grow throughout the brain tissue.** (A) Representative area sectioned (red lines) in a 7 dpt zebrafish. (B-F) Transverse 20-μm-thick cryosections of a GBM9 compact tumor at the level of the forebrain (B), midbrain (C,D) and hindbrain (E,F). (G-K) Transverse cryosections of a diffuse tumor at the level of the forebrain (G,H) midbrain (I) and hindbrain (J,K). (L) Based on morphology, tumors were scored as compact (light green bar) or diffuse (dark green bar) then measured by Sholl analysis at 7 dpt to quantify cell spread. Largest radius (in micrometers) is the measure of the farthest radius intersecting a cell from the injection site. *n*=10 per group; 20 animals total. *P<0.001. Scale bar: 40 μm for B-K.

Analysis of tumor morphology revealed that approximately 15% of animals developed compact tumors (Fig. 2A-A‴), whereas ∼85% of the fish developed diffuse tumors, with cells migrating away from the initial transplantation site (Fig. 2B-B‴). We did not see tumor cell spread until after 2 dpt, indicating that the transplant procedure itself was not diffusely distributing cells throughout the brain. Sholl analysis has been used previously to quantify stem cell migration (Imitola et al., 2004), and therefore we applied it here to quantify tumor cell spread. Confocal analysis of *z*-stacks from animals scored as having compact or diffuse tumors was carried out at 7 dpt. Sholl analysis was performed by placing the tracking ‘center’ dot at the flourescent tumor center as determined in compressed *z*-stack images and measuring the largest radius (enclosing radius) that tumor cells crossed. Data revealed that compact tumors lacked cell spread, whereas diffuse tumors had cells that migrated as far as 1.2 mm from the transplantation site. These data show that GBM9 cells can migrate widely within the zebrafish brain. To ensure that tumor cells were indeed penetrating into the brain, we examined 20 μm transverse sections for both compact (Fig. 3B-F) and diffuse tumors (Fig. 3G-K). We found GFP^+^ tumor cells located throughout the forebrain, midbrain and hindbrain. Whereas compact tumors showed large, continuous masses, diffuse tumors often had sections containing only a few cells at discontinuous sites (see Fig. 3J). Similar results were observed with X12 cells (data not shown). This provides further support for the migratory nature of GBM cells *in vivo*.

### Adherent and neurosphere glioma cell lines display unique characteristics *in vivo*

**Fig. 4.**
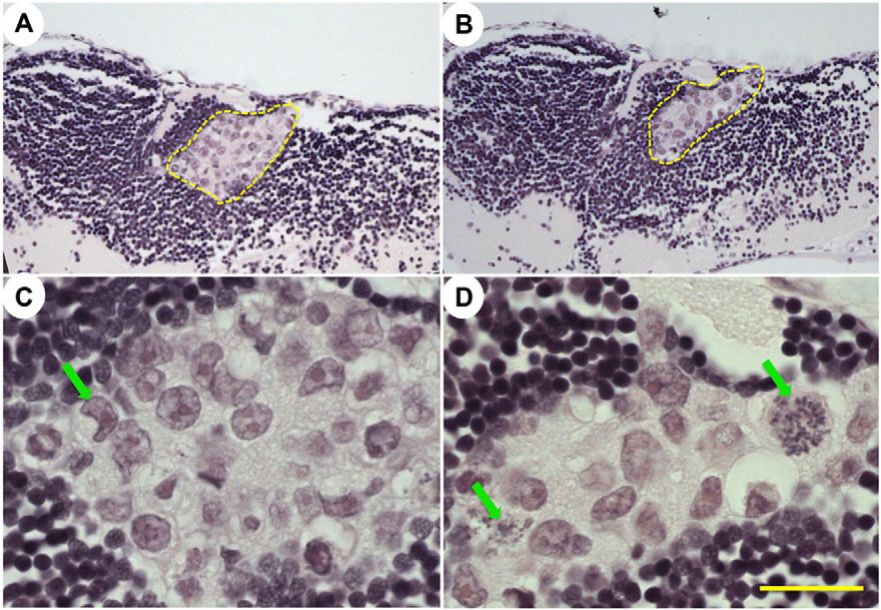
**GBM9 histology staining for Hematoxalin and Eosin.** (A-D) Paraffin-embedded GBM9 xenotransplanted animals at 7 dpt; 40× (A,B) and 100× (C,D) magnification of two separate animals (A,C and B,D) with tumors. Yellow dashed lines in A,B denote the tumor mass. Green arrows in C,D denote hyperchromatic and abnormal nuclei. Scale bar: 50 μm in A,B and 20 μm in C,D.

To analyze tumor morphology *in vivo*, we first performed Hematoxalin and Eosin staining at 7 dpt (Fig. 4). GBM9 tumors were clearly visible, with large cell nuclei and pink cytoplasm (yellow dashed outline in Fig. 4A,B). These tumors contained cells with abnormal nuclei (Fig. 4C, green arrow) and large, atypical mitoses (Fig. 4D, green arrows). This morphology is consistent with some of the key characteristics described in human GBM (Phillips et al., 2006).

**Fig. 5.**
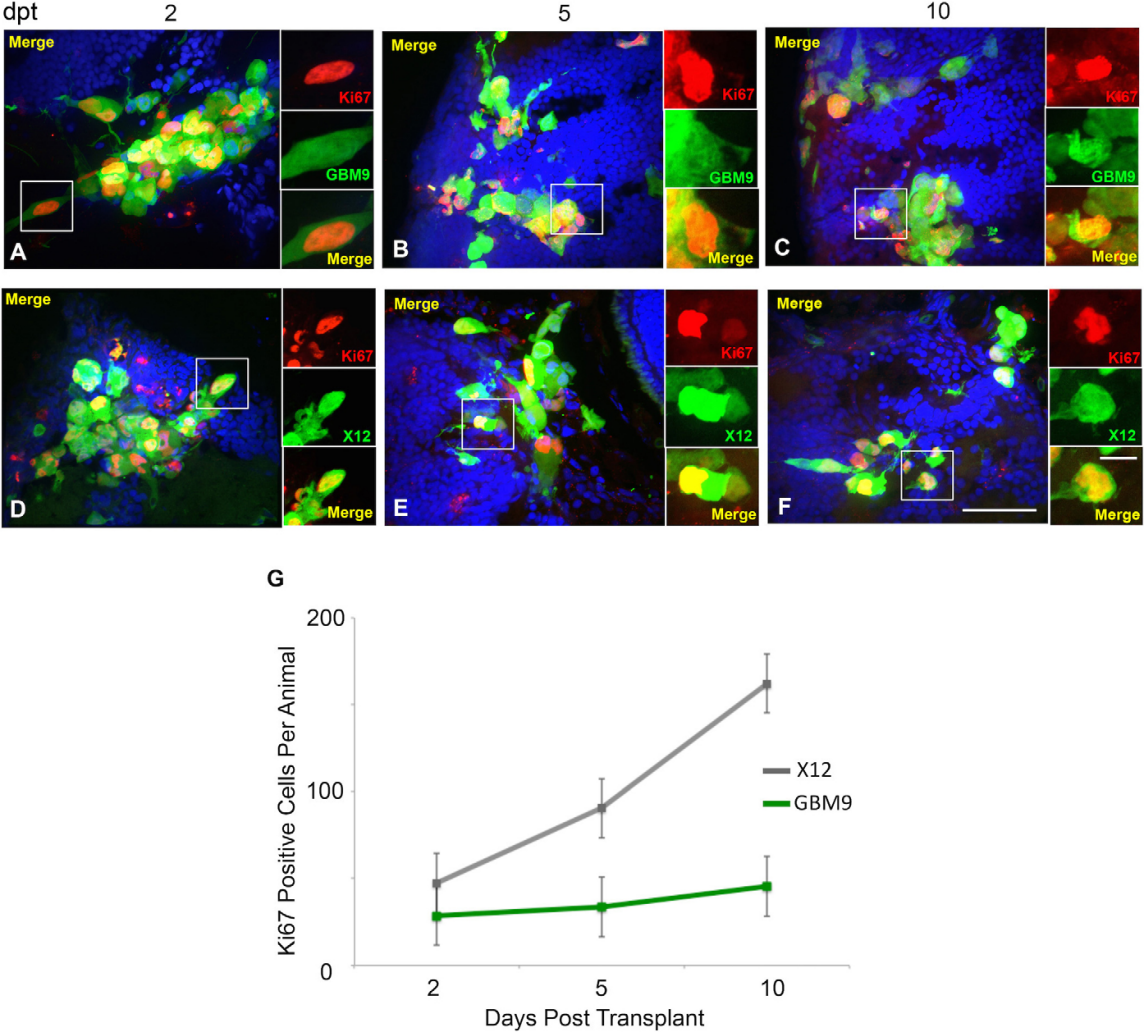
**GBM9 and X12 xenotransplants contain a high number of dividing cells.** (A-F) Confocal images of GBM9 and X12 on 2 (A,D), 5 (B,E) and 10 (C,F) dpt transverse cryosections. (A-C) GBM9 (green), DAPI (blue) and Ki67 (red) at 100×. (D-F) X12 (green), DAPI (blue) and Ki67 (red) at 100×. White boxes denote magnified area to the right of the image. *n*=5 animals per group; 30 animals total. Scale bar: 20 μm for main panels and 5 μm for insets. (E) Quantification of the total number of dividing cells per animal at each time point for GBM9 (green line) and X12 (gray line) transplants.

Immunohistochemistry was used to characterize these tumors further. For this analysis, we included both serum-grown adherent X12 cells and GBM9 cells to determine whether these tumors displayed unique characteristics *in vivo*. We first analyzed the expression of human Ki67, a marker of proliferation. The percentage of Ki67 cells was relatively constant (∼43%) for GBM9 tumors and was not statistically different between 2, 5 and 10 dpt (Table 1; Fig. 5A-C). In X12 tumors, the percentage of dividing cells was slightly higher and increased from 50 to 69% between 2 and 10 dpt, which was a statistically significant increase (Table 1; Fig. 5D-F). When examining the total number of Ki67^+^ cells per animal, X12 tumors had significantly more dividing cells at 10 dpt, presumably reflecting the greater number of cells in these tumors (Fig. 5G). Thus, both serum-grown adherent glioma cells and neurospheres contain a significant percentage of dividing cells *in vivo*.

**Table 1.**
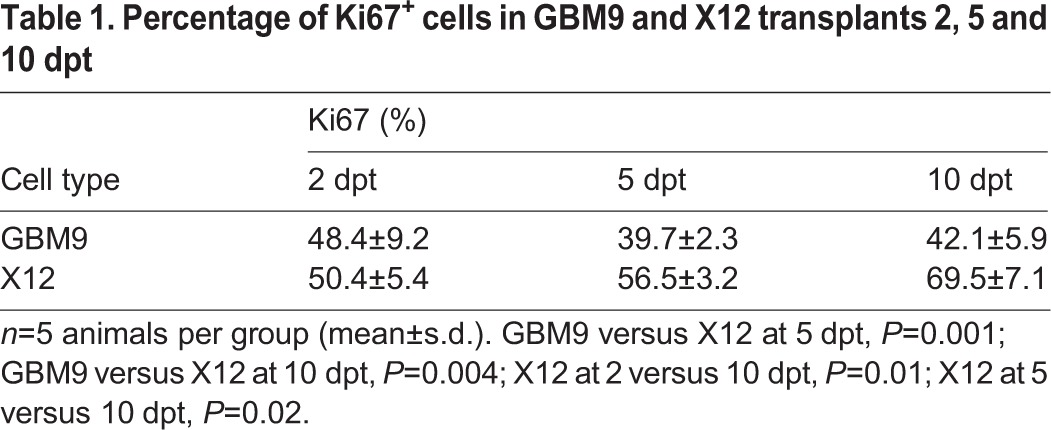
**Percentage of Ki67^+^ cells in GBM9 and X12 transplants 2, 5 and 10 dpt**

**Fig. 6.**
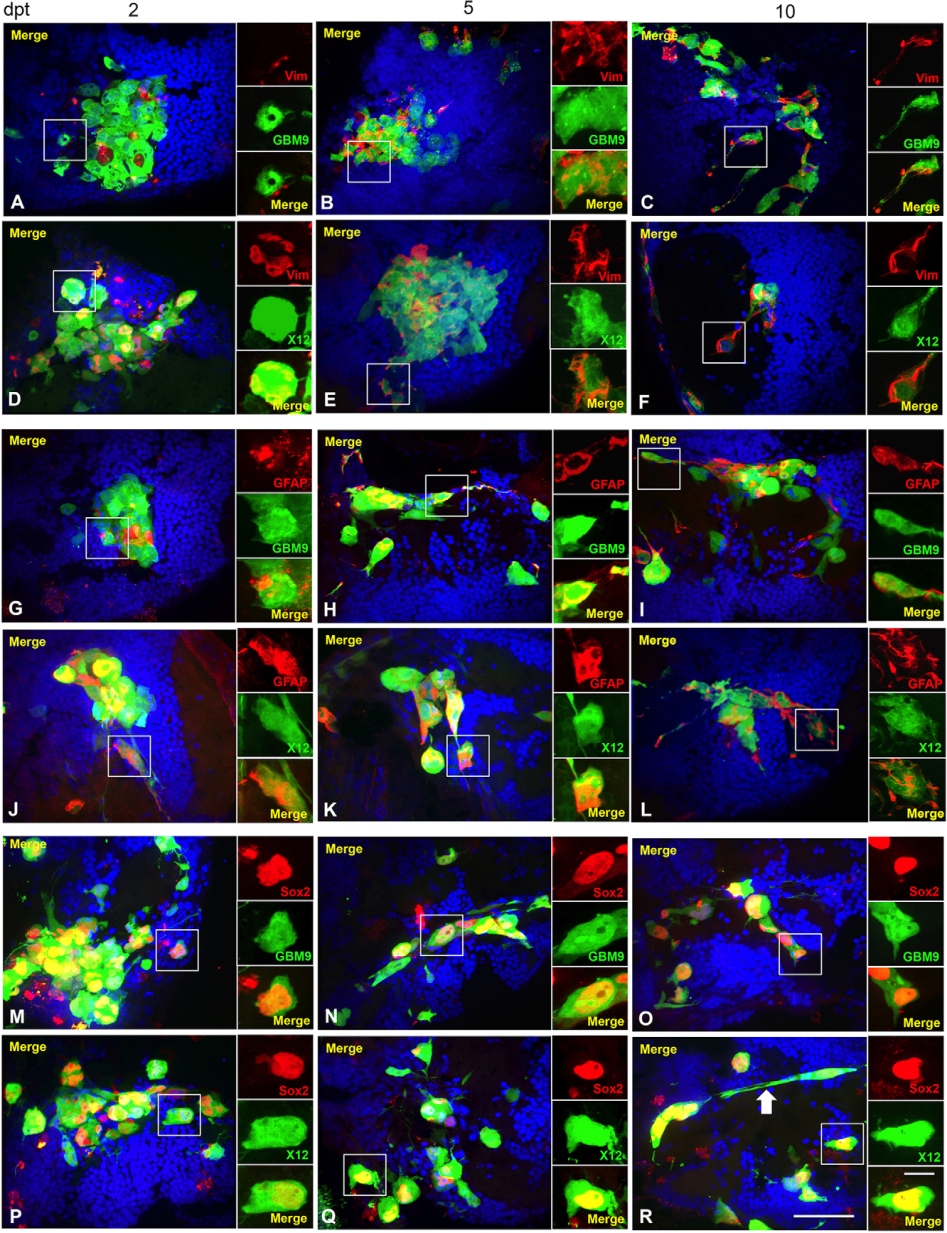
**GBM9 and X12 tumors contain a combination of differentiated cells and stem cells.** Confocal images of GBM9 and X12 on 2 (A,D,G,J,M,P), 5 (B,E,H,K,N,Q) and 10 (C,F,I,L,O,R) dpt transverse cryosections. (A-C) GBM9 (green), DAPI (blue) and vimentin (red) at 100×. (D-F) X12 (green), DAPI (blue) and vimentin (red) at 100×. (G-I) GBM9 (green), DAPI (blue) and GFAP (red) at 100×. (J-L) X12 (green), DAPI (blue) and GFAP (red) at 100×. (M-O) GBM9 (green), DAPI (blue) and Sox2 (red) at 100×. (P-R) X12 (green), DAPI (blue) and Sox2 (red) at 100×. White boxes denote area magnified to the right of the image. White arrow in R points to a cell with a migratory morphology. *n*=5 animals per group; 90 total animals. Scale bar: 20 μm for main panels and 5 μm for insets.

To determine whether the tumor cells were expressing markers of differentiation, we analyzed glial fibrillary acidic protein (GFAP) and vimentin at 2, 5 and 10 dpt. GFAP is an intermediate filament protein present in glial cells and is used as a marker of differentiation in human glioblastoma (Dunn et al., 2012). The cytoskeletal protein vimentin, expressed in normal human stem cells (Imitola et al., 2004), is overexpressed in GBM and is a marker of these cells (Colin et al., 2007). In GBM9 tumors at 2 dpt, both vimentin and GFAP were expressed at low levels (Table 2; Fig. 6A-C,G-I). There was a statistically significant increase in expression of both proteins between 2-5 and 5-10 dpt. These data support that GBM9 cells undergo differentiation in the zebrafish brain. By contrast, a high percentage of serum-grown X12 cells expressed GFAP and vimentin at 2 dpt, and these percentages remained high at 5 and 10 dpt (Table 2; Fig. 6D-F,J-L). The differentiated nature of these cells *in vivo* is consistent with what is observed for other serum-grown glioma cell lines in tissue culture (Gilbert and Ross, 2009). We also observed in both GBM9 and X12 transplants (white arrow in Fig. 6R) that many cells had trailing processes consistent with migrating neurons. These data show that glioma cells in the zebrafish brain retain characteristics consistent with their phenotype and that these neurosphere cells and adherent cell lines act differently *in vivo*.

**Table 2.**
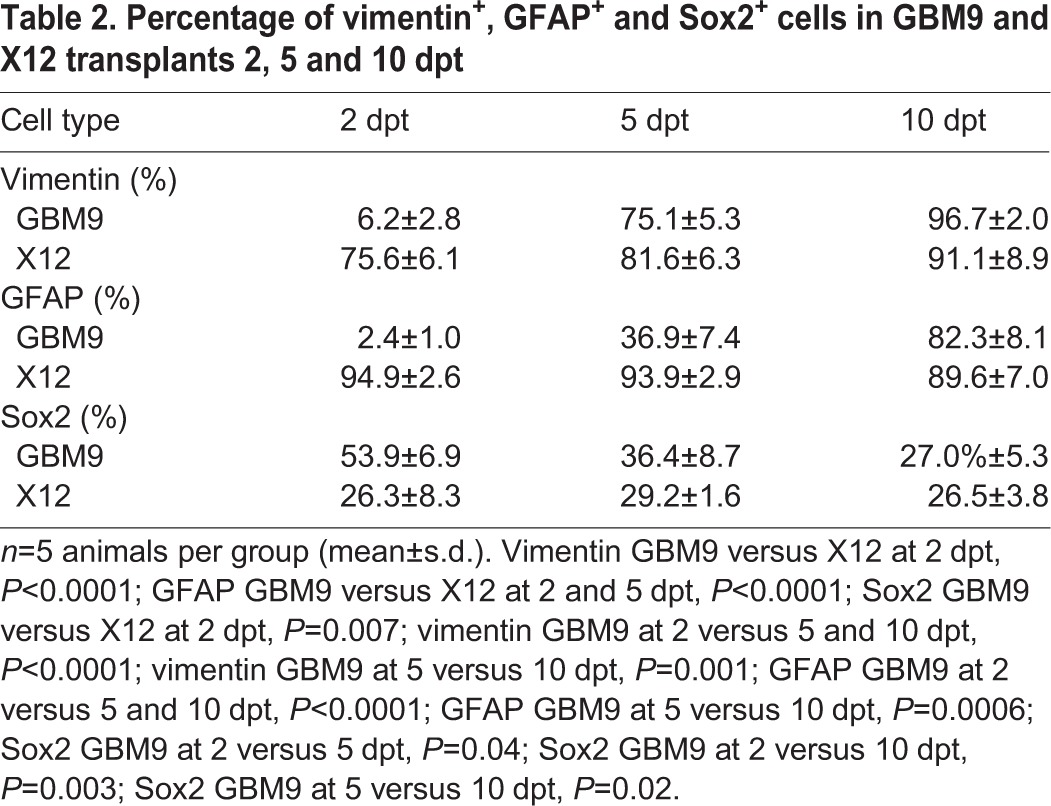
**Percentage of vimentin^+^, GFAP^+^ and Sox2^+^ cells in GBM9 and X12 transplants 2, 5 and 10 dpt**

Tumor stem cells are an important feature of patient tumors and are a presumed cause of recurrence (Dunn et al., 2012; Qiang et al., 2009). We therefore used a stem cell marker, Sox2 (Gilbert and Ross, 2009), to analyze stem cells in both GBM9 and X12 xenotransplants. In GBM9 transplants, ∼54% of cells were Sox2^+^ at 2 dpt (Table 2; Fig. 6M-O). This decreased significantly to 27% by 10 dpt. This might reflect the fact that GBM9 cells are becoming more differentiated during this time as revealed by the increase in vimentin and GFAP expression (Table 2). X12 cells were also Sox2^+^ *in vivo*, but with a constant percentage of cells (∼27%) expressing this marker from 2 to 10 dpt (Table 2; Fig. 6P-R). Thus, both serum-grown adherent cells and neurospheres contain Sox2^+^ cells indicative of stem cells, but the percentage of these cells is different as these populations of cells grow *in vivo*.

### Zebrafish GBM9 tumors are responsive to chemotherapeautic agents

**Fig. 7.**
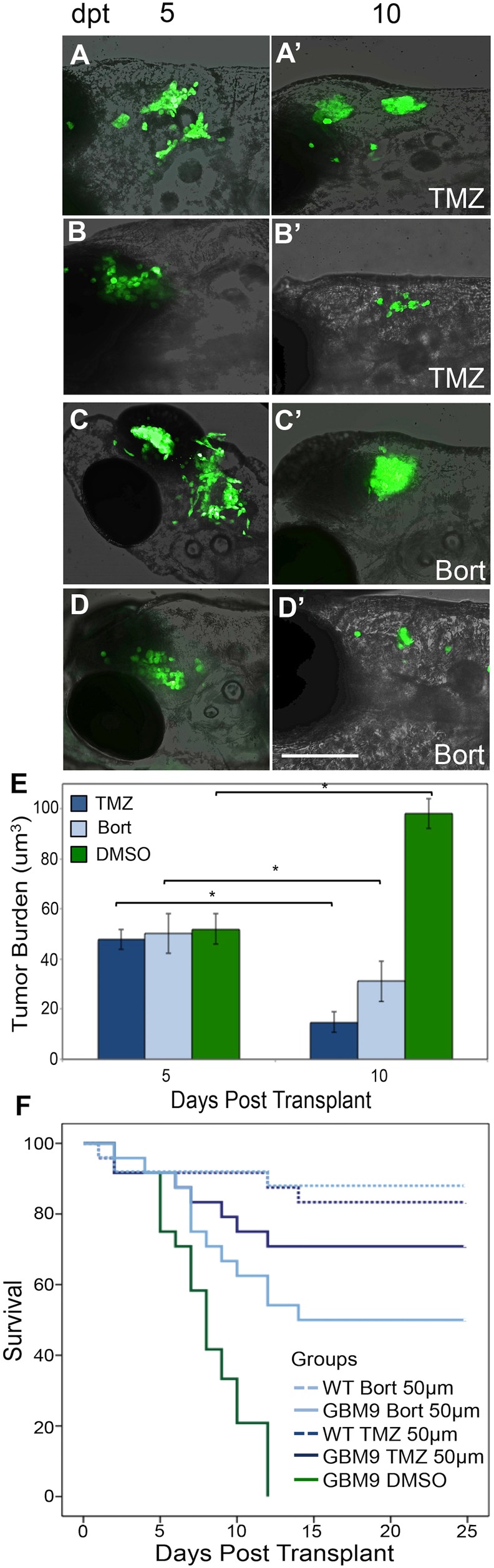
**Chemotherapeutic agents decrease GBM9 xenotransplant tumor burden.** GBM9 xenotransplants were treated with 50 μM drug continuously between 5 and 10 dpt. (A-D′) Confocal images superimposed on bright field (anterior to the left) of two GBM9 animals at 5 dpt (A,B) and at 10 dpt after 5 days of temozolomide (TMZ) treatment (A′,B′). (C,D) Confocal images superimposed on bright field (anterior to the left) of two GBM9 animals at 5 dpt (C,D) and at 10 dpt after 5 days of bortezomib (Bort) treatment (C′,D′). (E) Quantification of tumor burden (in micrometers cubed) before treatment (5 dpt) and after 5 days of treatment (10 dpt). *n*=10 animals per group. *P<0.001. (F) Kaplan–Meier survival curve of animals during drug treatment (5-10 dpt) with temozolomide (dark blue line) and bortezomib (light blue line). Control DMSO-treated GBM9 animals (green line) have a median survival of 8±0.6 days. Of the animals treated with TMZ, 70.8% lived until 25 days compared with 50.0% treated with bortezomib. Of the wild-type animals treated with 50 μm TMZ (dashed dark blue line) or bortezomib (dashed light blue line), 83.3 and 88.0%, respectively, survived. *n*=48 animals for all groups. *P*<0.0001 for GBM9 DMSO versus both GBM9 TMZ and GBM9 Bort. *P*=0.0672 for GBM9 TMZ versus GBM9 Bort. Scale bar: 50 μm.

Owing to the more aggressive and dynamic nature of the GBM9 neurospheres, we next asked whether these tumors were sensitive to the chemotherapeutic drugs temozolomide, which is DNA alkylating agent and FDA approved as a standard of care for GBM patients (Wesolowski et al., 2010), and bortezomib, a proteasome inhibitor used for multiple myeloma that is being tested in mouse models of glioblastoma (Styczynski et al., 2006). Given that tumor growth was robust during 5-10 dpt, we focused the drug treatment on this time period. We first performed a dose-response study on wild-type animals with doses based on Geiger et al. (2008). Temozolomide and bortezomib were tested separately in wild-type animals (24 animals per dose per compound) for toxicity at three doses (10, 50 and 100 μM) continuously from 6.5 to 11.5 days postfertilization (dpf), times consistent with 5-10 dpt at both 28° and 32°C. We found that these drugs were well tolerated at all of these doses, with ∼20-25% death at 50 μM, and thus we chose this dose because it had also been used previously in zebrafish (Geiger et al., 2008). Larvae transplanted with GBM9 cells were treated continuously from 5 to 10 dpt with 50 μM of either drug or 1% dimethyl sulfoxide (DMSO; Fig. 7A-D). Analysis of tumor burden in these fish at 10 dpt showed significant tumor reduction with either drug, although the response was more variable in bortezomib-treated animals (Fig. 7E). DMSO-treated (control) animals showed tumor growth as illustrated earlier (Fig. 2).

To determine whether decreased tumor burden was correlated with survival, animals were treated from 5 to 10 dpt with 50 μM TMZ or bortezomib and tracked until 25 dpt. Kaplan–Meier survival curves showed significant rescue of survival in GBM9 transplanted animals treated with either compound. Almost 80% of animals treated with temozolomide lived until 25 dpt, compared with 54% treated with bortezomib, whereas DMSO treatment did not affect survival (Fig. 7F; Table S1). These data show that GBM9 tumors in the zebrafish brain are responsive to chemotherapeutic agents.

## DISCUSSION

Although zebrafish have been used to analyze glioblastoma cell properties *in vivo*, these studies have used varying approaches and transplant paradigms, thus inhibiting the ability to compare data across experiments and laboratories. To increase the utility of this model, we provide a standardized approach and methods for tumor analysis *in vivo* that can be replicated easily in other laboratories, thus enhancing comparisons of GBM cells and drug treatments. Moreover, using this approach we show that glioma cells in the zebrafish brain display unique cellular characteristics *in vivo* and respond to chemotherapeutic treatments.

Studies have compared adherent glioma cell lines (U87MG and U251) with primary cultured neurosphere lines (GBM169 and U87MG in serum-free media) and found that the serum-free cultures produce more ‘human-like’ tumors *in vitro* and *in vivo* (Qiang et al., 2009; Galli et al., 2004). Transplantation of neurospheres into nude mice replicates many features of human glioblastomas, such as histopathology (pseudopalisades and necrosis), cellular characteristics (differentiation and invasion) and chromosomal aberrations typical of patient tumors (EGFR activation and telomerase re-activation) compared with the serum-grown U87 counterparts, which show none of these characteristics (Molina et al., 2014). Tumor stem cell populations are thought to be essential to tumor formation and recurrence of glioblastoma (Chaffer and Weinberg, 2015). GBM9 neurospheres in the zebrafish brain show many of these same characteristics, supporting the utility of this model. They have high proliferative capacity, as shown by Ki67, and the level of proliferation remains relatively constant from 2 to 10 dpt. Interestingly, these cells are very undifferentiated at early time points (2 dpt) but become more differentiated over time *in vivo*, and by 10 dpt they are highly differentiated. A reciprocal pattern is seen with the stem cell marker Sox2. At 2 dpt, more than 50% of GBM9 cells express Sox2, and this decreases significantly by day 10 to 27%. This pattern suggests that GBM9 cells are dynamic in the zebrafish brain and become more differentiated, while still retaining a stem cell population, as the tumors develop over 8 days *in vivo*. Having a dynamic Sox2 population makes this an excellent model in which to test the effect of drugs on the tumor stem cell population. Serum-treated adherent X12 cells in the zebrafish brain, by contrast, expressed differentiated characteristics at the first time point examined. For example, ∼95% of X12 cells expressed GFAP at 2 dpt compared with ∼2% of GBM9 cells at this same stage. This high level of X12 differentiation was maintained, as defined by GFAP and vimentin expression. These data showing that serum-grown X12 cells were more differentiated *in vivo* are consistent with *in vitro* data (Lee et al., 2006) and mouse data (Suva et al., 2014). However, even though the X12 cells were more differentiated, they still contained a population of Sox2^+^ cells and formed tumors that led to early lethality in zebrafish. Interestingly, a recent study maintained human GBM patient cells either in serum or as neurospheres and found that only the neurospheres generated tumors when transplanted into mice (Suva et al., 2014). Although X12 cells did generate tumors, as did another adherent cell line, U87 (Lal et al., 2012), in zebrafish brains, this is consistent with our finding that fewer GBM9 cells were needed and lethality was greater compared with X12 cells. One explanation for this might be the higher initial population of Sox2^+^ stem cells in the GBM9 transplants.

Analysis of Ki67 labeling showed that both neurospheres and adherent cells underwent cell division in the zebrafish brain. The percentage of dividing cells did not significantly change for GBM9 cells, staying around 43%, but the percentage of dividing cells in X12 tumors increased over the 8 days of analysis from 50 to ∼70%. In terms of the number of dividing cells, it was 2.7- and 3.6-fold higher in X12 transplanted animals at 5 and 10 dpt, respectively, compared with GBM9 tumor-containing animals (see Fig. 5G). It is important to note, however, that twice as many X12 cells were transplanted. We cannot say for certain why the less aggressive cell type had a higher number of dividing cells. This suggests, however, that other cellular characteristics besides the capacity to divide are determining aggressiveness. For example, GBM9 tumors start out having a higher percentage of Sox2-expressing cells and fewer differentiated cells. Perhaps this represents a more aggressive tumor cell population then in the X12 transplants, where the majority of cells differentiate soon after transplantation.

Although glioma is usually an adult-onset cancer, there are benefits to generating a larval zebrafish model of this and other cancers (White et al., 2013). Larval zebrafish have an innate, but not an acquired/adaptive, immune system (Taylor and Zon, 2009). This is beneficial in two ways: the animals do not need to be immunocompromised before transplantation, and a part of the immune system is still active. The zebrafish innate immune system is present from an early larval stage, but the acquired immune system does not develop until ∼28 dpf (Rauta et al., 2012). This allows for transplantation of human cells without immune rejection, but retains an aspect of the immune system that might be functioning in the host response to tumor cells, creating a more relevant tumor microenvironment.

Another major benefit of using larval zebrafish is the ability to perform *in vivo* cell analysis. The zebrafish larval brain is very amenable to live imaging facilitated by mutant lines, such as *casper*, that are transparent because of mutations in pigment genes, thus allowing direct visualization of tumor cells in the brain. These animals not only allow for visualization of single cells, but also the ability to image the same tumor over time because the animals do not need to be sacrificed for tumor analysis. Using Sholl analysis and MetaMorph software for cell tracking, we have successfully quantified tumor spread *in vivo*. Interestingly, we found that the vast majority of tumors (∼83%) were diffuse and composed of cells that had migrated away from the initial transplantation site, spreading in both anterior and posterior directions within the brain. We did not, however, find tumor cells in the spinal cord. Using a controlled pressure injector allowed us to rule out cell spread caused by the transplantation procedure. Indeed, we found only compact tumors at 2 dpt, and diffuse tumors were present at 5 dpt, indicating that this property emerged over time (see Fig. 2). Lal et al. (2012) also described glioma cell migration in the zebrafish brain and showed that this migration was inhibited by decreasing calpain 2, a calcium-activated protease. We also observed in both the GBM9 and X12 transplants that by 5 dpt, many cells had an elongated bipolar morphology consistent with migrating neurons (O'Rourke, 1996; Massalini et al., 2009). Given that these cells are not migrating along radial glia and appear to be growing along one another, it is reminiscent of chain migration (O'Rourke, 1996); however, whether this is the primary mechanism of neuronal migration in these transplants remains to be determined. These data support the suggestion that serum-grown adherent glioma cells and neurospheres migrate within the zebrafish brain and that the dispersion seen in these models is the result of active tumor cell migration.

Perhaps the major benefit of using larval zebrafish, however, is the ability to perform whole-vertebrate animal drug screens. This is because of the large number of larval animals that can be screened in 96-well plates and the excellent *in vivo* imaging capability (White et al., 2013). A number of the parameters defined here can be used to measure drug efficacy; these include tumor burden, cell migration, cell proliferation, cell differentiation and stem cell population. New drug compounds have already been identified in zebrafish models of cancer that have led to clinical trials in patients (White et al., 2013), supporting the utility of this approach. As a proof of principle that this model can be used for drug screening, we tested two chemotherapeutic agents and found that they reduced tumor burden and increased survival post tumor transplant. Future studies using temozolomide in combination with new agents will allow for synergistic experiments that directly mimic combinations that patients will be exposed to in the clinical situation. Compounds that act as anti-tumor agents in cell culture can be tested in this model first for toxicity and for tumor ablation. Top hits from these studies can then move into murine models instead of screening a large number of compounds in mice initially, thus reducing the cost and time of the drug screen. Another exciting future direction would be to create individualized models of GBM by taking resected tumors from patients, culturing neurospheres, labeling the cells and transplanting them into the zebrafish brain to test specific therapeutics, thereby creating a personalized model for glioma (Veinotte et al., 2014). Thus, characterization and standardization of a glioma model in zebrafish has the potential to have a high impact, especially for a disease that has a high mortality rate and few therapeutic options.

## MATERIALS AND METHODS

### Cell culture

GBM9 and X12 cells were obtained from tumor specimens as previously described and modified with GFP to generate GBM9-GFP and X12-v2 (Williams et al., 2011; Godlewski et al., 2008; Wojton et al., 2014; Giannini, 2005). Neurospheres (GBM9, mNSC) were kept in Neurobasal Media (Gibco, Grand Island, NY, USA) supplemented with B-27, GlutaMax (Gibco) and growth factors EGF and FGF (R&D Systems, Minneapolis, MN, USA) in flasks (Corning Inc., Corning, NY, USA). Adherent cells (X12-v2) were kept in Dulbecco's modified Eagle's medium (Gibco) and 10% fetal bovine serum for the first week in culture and then in 2% fetal bovine serum for the duration of experiments (GE Healthcare, Logan, UT, USA) in culture dishes (Corning Inc.). Cells were tested for mycoplasma by PCR every 8-12 weeks (GBM9 neurospheres) or 6 months (X12 cells). Both lines were GFP labeled and are referred to throughout this work as GBM9 and X12.

### Zebrafish lines

Zebrafish were maintained at 28°C unless otherwise noted. AB×Tupfel long fin (ABLF) animals are referred to as wild type. *casper* mutants (*roy*;*nacre*; White et al., 2008) were obtained from Dr Leonard Zon's laboratory at Children's Hospital Boston. All animals were kept in accordance with The Ohio State University Institutional Animal Care and Use Committee protocols. For all experiments, animals were obtained from group crosses.

### Transplants

For transplantation, X12 cells were grown to 70-80% confluence then washed twice with PBS (Gibco), trypsinized (Gibco), dissociated, counted, and resuspended in Hank's balanced salt solution (HBSS; Gibco). When GBM9 neurospheres reached ∼1 mm diameter they were dissociated using TrypLE (Gibco). GBM9 cells were counted and resuspended in HBSS (Gibco) within 15 min of transplantation. GFP-labeled mNSC cells were obtained from Dr Jaime Imitola's laboratory and counted for control experiments. Cells were transplanted in the vicinity of the midbrain-hindbrain boundary of 36 hpf tricaine-anesthetized embryos (Sigma-Aldrich, St Louis, MO, USA) using a back-loaded pulled borosilicate glass needle (Sutter Instruments, Novato, CA, USA) and avoiding the ventricle. Sham-injected animals received an injection of 1-2 nl of HBSS into the midbrain. After transplantation, animals were allowed to recover in fish water and 100 units penicillin/100 µg streptomycin/ml (Invitrogen, Grand Island, NY, USA) in 24-well plates (Corning Inc.) on a warming plate at 32°C. For tumor cell engraftment, animals were screened at 24 h post-transplantation (hpt) and, based on cell counts, fell into one of the following four groups: (i) 10-25 cells; (ii) 26-50 cells; (iii) 51-90 cells; or (iv) 91-140 cells. Animals were then tracked for survival. After the optimal cell number was established, larvae were screened at 24 hpt for ∼50 cells for GBM9 and ∼100 cells for X12 per animal. Animals without the optimal number of cells were screened out of the study. SPSS (Statistical Package for the Social Sciences; IBM Corp., Armonk, NY, USA) was used to create Kaplan–Meier survival curves and to calculate median survival. Animals were fed, beginning at 6 dpf, using standard larval food. Small amounts of food were added to each 24-well plate and rinsed out after 30 min with fresh fish water. Experiments consisted of animals obtained from multiple group crosses (usually three females and two males) and cells obtained from the same passage. Animals was obtained from between two and four experiments consisting of ∼24 transplants per experiment. Details on numbers of animals are provided in the figure legends.

### Tumor burden analysis

Individual tumor-bearing or mNSC-transplanted animals were anesthetized using tricaine (160 μg/ml) at 2, 5, 7 and 10 dpt and imaged in a FluoroDish (WPI, Inc., Sarasota, FL, USA) under the spinning disk confocal microscope (Andor). Using MetaMorph software (Molecular Devices, Sunnyvale, CA, USA) and Fiji (Schindelin et al., 2012), images captured through the zebrafish tumors were reconstructed using a *z*-stack to analyze tumor volume. Using MetaMorph, a standard cell volume in fluorescent pixels was averaged from six cells. This ‘standard’ cell volume was then applied to the entire *z*-stack. These measurements were converted into volume (in micrometers cubed) using a MetaMorph software formula that converts a standardized pixel analysis of one ‘cell’ into a volumetric number. Images were analyzed using Fiji/ImageJ and Excel. The same animal was imaged on 2, 5, 7 and 10 dpt, and tumor burden was quantified and plotted for each of these days using Excel.

### Sholl analysis

A modified Sholl analysis was carried out using Fiji (Schindelin et al., 2012). Using the 7 dpt compressed *z*-stack images of the transplanted animals, a tracking ‘center’ dot was placed in the center of the fluorescent tumor, which is where the cells were originally transplanted. The largest radius (enclosing radius) that tumor cells crossed was measured.

### Cryostat sections

GBM9- and X12-transplanted animals were fixed at 2, 5, 7 or 10 dpt with 4% paraformaldehyde (PFA; Sigma-Aldrich) in PBS (Sigma-Aldrich) at 4°C. At least 24 h was necessary for fixation because tumor cells are softer than the surrounding fish brain. Larvae were then transferred into 30% sucrose in PBS (Thermo-Fisher Scientific, Waltham, MA, USA) at 4°C overnight, then placed individually into silicone molds and frozen in OCT compound (Sakura Finetek, Torrance, CA, USA). Animals were sectioned at 20-25 µm on a cryostat machine and sections collected on Super Frost Plus slides (Thermo-Fisher Scientific). Slides were subsequently used for histology and immunostaining. Sections were cut in the transverse orientation, which is the same orientation as coronal brain sections in the mouse and human.

### Histology and immunohistochemistry

#### Hematoxalin and Eosin

For Hematoxalin and Eosin staining, animals at 7 dpt were fixed in 4% PFA (Sigma-Aldrich) in PBS (Gibco, Grand Island, NY, USA). They were then paraffin embedded, sectioned and stained with Hematoxalin and Eosin by the Ohio State Comparative Pathology Laboratory, College of Veterinary Medicine, Columbus, OH, USA.

All staining was performed on 20 μm cryostat sections. Primary antibodies were diluted in 3% bovine serum albumin in PBS and incubated at 4°C overnight. All secondary antibodies were diluted in 0.1% Triton in PBS and incubated for 2 h at room temperature.

#### Ki67

After cryosection, slides were lined with a Dako Pen (Dako, Carpinteria, CA, USA). Slides were washed for 30 min in PBS, then switched into 0.5% Triton (Thermo-Fisher Scientific) in PBS for 10 min. Slides were then washed for 10 min in PBS. Antigen retrieval was performed by placing slides in boiling PBS for 14 min. Slides were blocked in 3% bovine serum albumin (Jackson ImmunoResearch, West Grove, PA, USA) for 2-4 h, then incubated in anti-Ki67 (D3B5) rabbit antibody (1/100; Cell Signaling, Danvers, MA, USA; 9129S) at 4°C overnight. Subsequently, slides were washed for 1 h in PBS, then switched into 0.1% Triton for 10 min. Slides were placed into secondary antibody (Life Technologies, Carlsbad, CA, USA) Alexa-Fluor 594 goat anti-rabbit IgG (1/200) for 2 h at room temperature. Slides were finally washed for 1 h in PBS, mounted using Fluoromount with 4′,6-diamidino-2-phenylindole dihydrochloride (DAPI; Sigma-Aldrich) and coverslipped for imaging (Thermo-Fisher Scientific).

#### Vimentin

The same procedure as for Ki67 was followed, but without antigen retrieval. Vimentin primary monoclonal antibody (mouse clone V9, 1/200; Dako; M0725) and secondary antibody Alexa-Fluor 594 rabbit anti-mouse IgG (1/200; Life Technologies) were used.

#### GFAP

Methods were identical to Ki67 staining above, but without antigen retrieval. The primary antibody was polyclonal rabbit anti-glial fibrillary acidic protein (GFAP, 1/200; Dako; Z0334), and secondary antibody was Alexa-Fluor 594 goat anti-rabbit IgG (1/200) as above.

#### Sox2

Methods were identical to Ki67 staining above with antigen retrieval. The primary antibody was polyclonal rabbit anti-Sox2 antibody (1/50; Abcam, Cambridge, MA, USA; ab97959), and secondary antibody was Alexa-Fluor 594 goat anti-rabbit IgG (1/200) as above.

### Drug screening

Compounds temozolomide (Sigma-Aldrich; T2577) and bortezomib (LC Laboratories, Woburn, MA, USA; B-1408) were tested in wild-type ABLF animals (24 per dose per compound; six groups of 24) for toxicity at three doses (10, 50 and 100 μM) from 6.5 to 11.5 dpf at both 28 and 32°C. Survival curves were plotted and a 50 μm dose was determined based on this toxicity analysis plus data from Geiger et al. (2008). Toxicity was the same for the two temperatures. Animals at 36 hpf were transplanted with GBM9 cells as described. Animals at 5 dpt (comparable to 6.5 dpf) were placed into one of the following three groups: 1 ml 1% DMSO (Thermo-Fisher Scientific) in fish water; 1 ml 50 μm temozolomide in fish water; or 1 ml 50 μm bortezomib in fish water. Animals were treated for 5 days (from 5 to 10 dpt) at 32°C in 24-well plates. Fish water and drugs were changed every day of the 5 days of treatment. Animals were imaged on the spinning disk confocal microscope at 5 dpt before treatment and then again at 10 dpt. Tumor burden was quantified using methods outlined above. Animals were then fixed in 4% PFA overnight for sectioning. In separate studies, animals were treated as above and tracked for survival, which was quantified using Kaplan–Meier survival curves (SPSS; IBM Corp.).

### Imaging

Imaging for analysis of tumor burden in live zebrafish was conducted on the Andor spinning disk confocal microscope (Andor, Oxford, UK; Figs 2 and 7). Cryostat section analysis was performed on the Zeiss (Oberkochen, Germany) Axioplan microscope (Fig. 3). Paraffin sections were analyzed on a Zeiss Axioplan microscope with differential interference contrast optics (Fig. 4). Immunohistochemistry was imaged on the Andor spinning disk confocal microscope. *Z*-stacks of 0.4 μm were obtained through 20-μm zebrafish sections and imaged with filters 405 (blue; DAPI), 488 (green; tumor cells) and 594 (red; immunohistochemistry). Stacks were then compressed in the 488 and 594 channels to create one image. Compressed stacks from 488 and 594 channels were overlaid with the 405 plane to create merged images. DAPI (blue) was used as a reference point, and thus only one plane per stack was used in the overlay. Images from 488 and 594 channels were cropped for the insets and overlaid for the merge to show staining details (Figs 5 and 6).

### Statistical analysis

For survival curves (Fig. 1; Fig. 7F), significance was determined by a log-rank test. For Table 1 (data for Figs 5 and 6), Fig. 3L (tumor morphology) and Fig. S1 (evoked swimming), *P*-values were determined by two-tailed Student's unpaired *t-*test. Fig. 7E (tumor burden) *P*-values were determined by two-tailed Student's paired *t-*test.

### Touch-evoked swimming assay

Wild-type (ABLF), sham-injected or GBM9-transplanted animals on 5 and 10 dpt were placed into the center (indicated by a black dot) of a 10 cm Petri dish filled with fish water. Animals were then poked in the trunk using a fly-pin, and the distance they moved was quantified. The final radius of the swim (as indicated by where the animal landed in circles that increased by 1 cm drawn on the bottom of the Petri dish) was plotted on a bar graph as distance traveled (in centimeters). This is presented in Fig. S1.
