# Correction: Standardized orthotopic xenografts in zebrafish reveal glioma cell-line-specific characteristics and tumor cell heterogeneity

**DOI:** 10.1242/dmm.027235

**Published:** 2016-09-01

**Authors:** Alessandra M. Welker, Brian D. Jaros, Vinay K. Puduvalli, Jaime Imitola, Balveen Kaur, Christine E. Beattie

There were errors published in three figures in *Dis. Model. Mech*. **9**, 199-210.

Fig. 2, Fig. 3 and Fig. 7: The scale bars in the confocal images were incorrectly depicted and described. In the amended figures below correct scale bars have been given and defined in the legends.

**Fig. 2.**
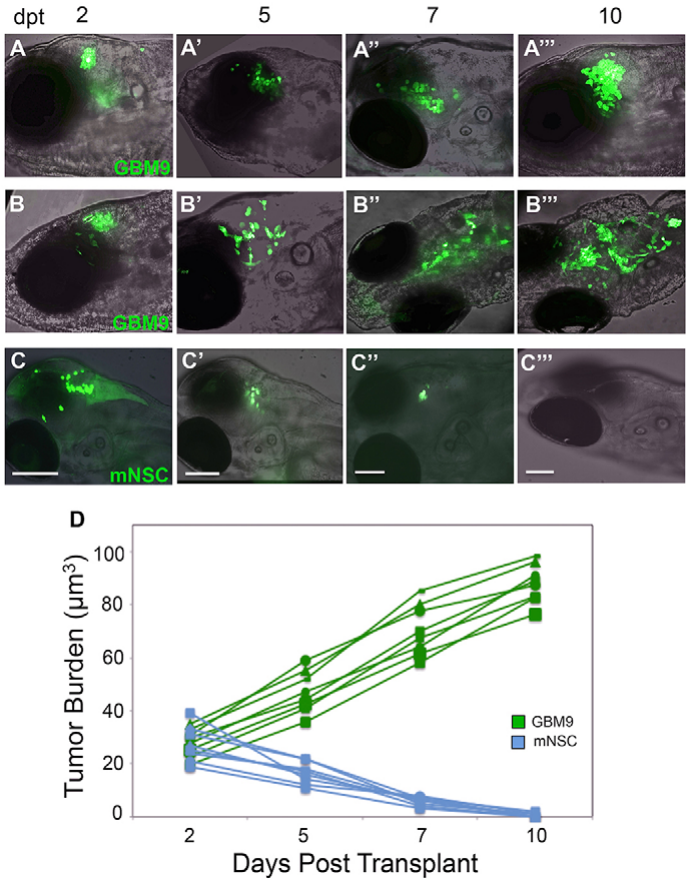
**Analysis of tumor burden in live animals over time.** Confocal images superimposed on bright field (anterior to the left) of two representative *casper* zebrafish transplanted with 50-75 GBM9 cells (A-A‴,B-B‴) and a *casper* animal transplanted with control mNSC cells (C-C‴) imaged at 2 (A,B,C), 5 (A′,B′,C′), 7 (A″,B″,C″) and 10 (A‴,B‴,C‴) dpt. Examples of a compact (A-A‴) and diffuse tumor (B-B‴) are shown. (D) Tumor burden were quantified using volume measurements of florescence in micrometers cubed. Approximately 50-75 GBM9 cells (green lines) and ∼50 mNSC cells (blue lines) were transplanted and followed over time in the same animal. *n*=8 animals per group. Scale bars: 100 μm.

**Fig. 3.**
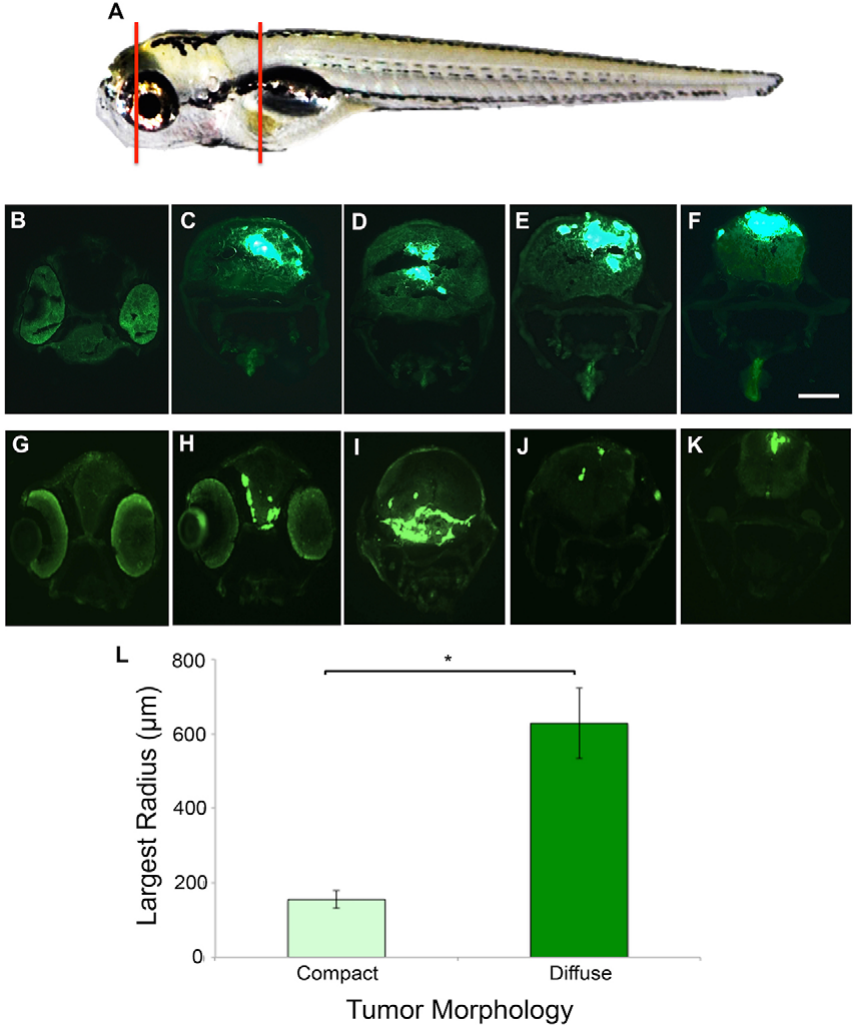
**GBM9 tumor cells grow throughout the brain tissue.** (A) Representative area sectioned (red lines) in a 7 dpt zebrafish. (B-F) Transverse 20-μm-thick cryosections of a GBM9 compact tumor at the level of the forebrain (B), midbrain (C,D) and hindbrain (E,F). (G-K) Transverse cryosections of a diffuse tumor at the level of the forebrain (G,H) midbrain (I) and hindbrain (J,K). (L) Based on morphology, tumors were scored as compact (light green bar) or diffuse (dark green bar) then measured by Sholl analysis at 7 dpt to quantify cell spread. Largest radius (in micrometers) is the measure of the farthest radius intersecting a cell from the injection site. *n*=10 per group; 20 animals total. *P<0.001. Scale bar: 100 μm for B-K.

**Fig. 7.**
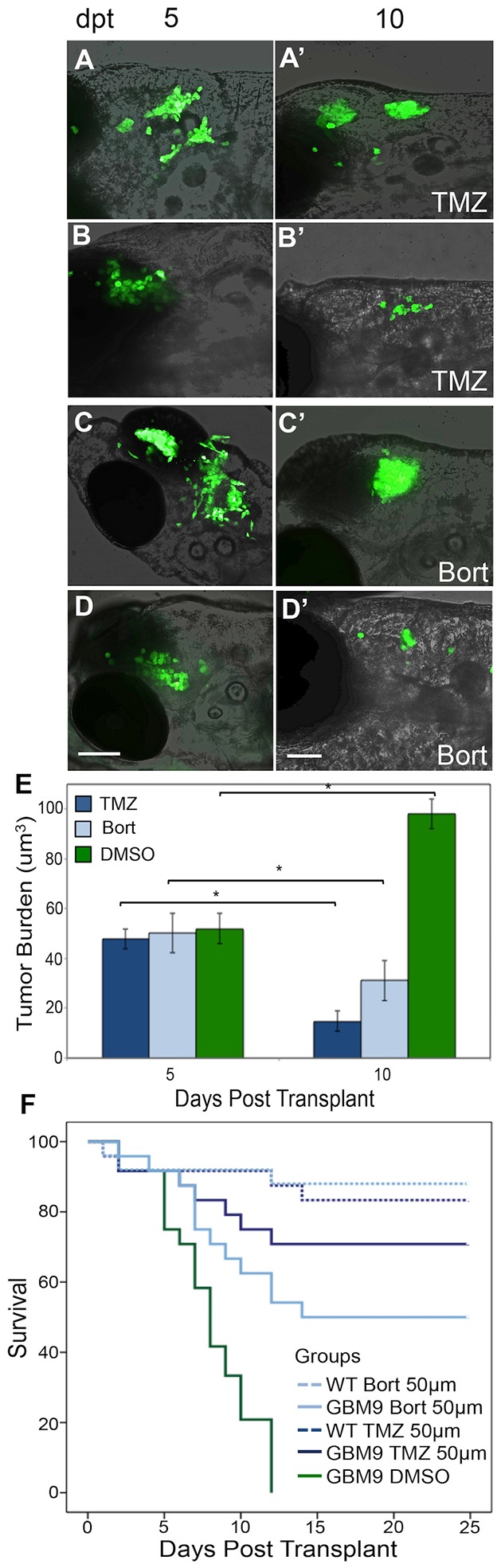
**Chemotherapeutic agents decrease GBM9 xenotransplant tumor burden.** GBM9 xenotransplants were treated with 50 μM drug continuously between 5 and 10 dpt. (A-D′) Confocal images superimposed on bright field (anterior to the left) of two GBM9 animals at 5 dpt (A,B) and at 10 dpt after 5 days of temozolomide (TMZ) treatment (A′,B′). (C,D) Confocal images superimposed on bright field (anterior to the left) of two GBM9 animals at 5 dpt (C,D) and at 10 dpt after 5 days of bortezomib (Bort) treatment (C′,D′). (E) Quantification of tumor burden (in micrometers cubed) before treatment (5 dpt) and after 5 days of treatment (10 dpt). *n*=10 animals per group. *P<0.001. (F) Kaplan–Meier survival curve of animals during drug treatment (5-10 dpt) with temozolomide (dark blue line) and bortezomib (light blue line). Control DMSO treated GBM9 animals (green line) have a median survival of 8±0.6 days. Of the animals treated with TMZ, 70.8% lived until 25 days compared with 50.0% treated with bortezomib. Of the wild-type animals treated with 50 μm TMZ (dashed dark blue line) or bortezomib (dashed light blue line), 83.3 and 88.0%, respectively, survived. *n*=48 animals for all groups. *P*<0.0001 for GBM9 DMSO versus both GBM9 TMZ and GBM9 Bort. *P*=0.0672 for GBM9 TMZ versus GBM9 Bort. Scale bars: 100 μm.

Fig. 3: In panel L (Sholl analysis), the *y*-axis label values were incorrect; the amended panel is included in the figure is below. There are no further changes to the figure legend beyond the change to the scale bar described above; the legend was otherwise accurate.

These errors do not affect any significance measures nor do they alter any conclusions of this paper.

The authors apologise to the readers for any confusion that these errors might have caused.

